# Future Incidence of Malignant Mesothelioma in South Korea: Updated Projection to 2038

**DOI:** 10.3390/ijerph18126614

**Published:** 2021-06-19

**Authors:** Kyeongmin Kwak, Sung-il Cho, Domyung Paek

**Affiliations:** 1 Department of Occupational and Environmental Medicine, Korea University Ansan Hospital, Ansan 15355, Korea; ggm1981@snu.ac.kr; 2 Department of Public Health Sciences, Graduate School of Public Health, Seoul National University, Seoul 08826, Korea; scho@snu.ac.kr; 3 Institute of Health and Environment, Seoul National University, Seoul 08826, Korea; 4 Department of Environmental Health Sciences, Graduate School of Public Health, Seoul National University, Seoul 08826, Korea

**Keywords:** mesothelioma, malignant mesothelioma, asbestos, prediction model, age-period-cohort model, NORDPRED, Poisson regression

## Abstract

Malignant mesothelioma (MM) is a cancer that is largely caused by exposure to asbestos. Although asbestos is no longer used in South Korea, the incidence of MM continues to increase due to its long latent period. We aimed to update the previous prediction of MM incidence until 2038. We predicted the incidence of MM over the next 20 years (2019–2038) in South Korea using Møller’s age–period–cohort (APC) model and a Poisson regression model based on asbestos consumption. The APC model predicted that the crude incidence rate would increase sharply in men and slowly in women. Despite the sex discrepancy in the rate of increase, the incidence rate for both sexes is expected to continue increasing until 2038. In the Poisson model, the crude incidence rate was predicted to increase continuously until 2038, and far more cases of MM were predicted to occur compared with the results of the APC model. When compared with actual incidence data, the APC model was deemed more suitable than the Poisson model. The APC model predicted a continuous increase over the next 20 years with no peak, suggesting that the incidence of MM will continue to rise far into the future.

## 1. Introduction

Malignant mesothelioma (MM) is a type of cancer that develops in the mesothelium of the pleura or peritoneum, with more than 80% of cases caused by asbestos exposure [1]. MM is a rare and aggressive cancer with a low survival rate. The latent period is very long, ranging from 30 to 40 years [2].

In South Korea, asbestos use peaked in 1992 and began to decline sharply after the use of amosite and crocidolite was banned in 1997 [3]. In 2009, all types of asbestos were banned. However, due to the long latent period, diagnoses of MM continue to increase. In 2017, we estimated the future incidence of MM from 2014 to 2033 using Møller’s age–period–cohort (APC) model. The future incidence was estimated to increase continuously until 2033 in men and until 2024–2028 in women, decreasing thereafter [4]. However, no information on asbestos use was included, and no validation was performed through comparison with other models. Although many countries have banned or restricted the use of asbestos, the global incidence of MM is increasing. The global burden of MM increased from 372,112 disability-adjusted life years (DALY) in 2000 to 586,970 DALY in 2019 [5]. According to a study by the Health and Safety Executive, the economic burden of MM in the United Kingdom (UK) caused by asbestos was GBP 3 billion, accounting for a significant proportion of the GBP 12.3 billion economic burden of occupational cancer [6,7]. Therefore, MM, which is typically caused by asbestos, is an important public health issue.

For this reason, since the 1990s, several studies, mainly in Europe and the United States (US), have aimed to predict MM incidence and mortality. In 1995, using an age–cohort model, Peto et al. projected that the mortality rate of MM in the UK would peak in 2020 [8]. Ilg et al. predicted MM mortality in France from 1996 to 2020 using an age–cohort model, as in the UK study, and found that deaths from MM would continue to increase until 2020 [9]. Kjærgaard et al. predicted the incidence of MM in Denmark using an age–cohort model and predicted a peak around 2015 [10]. Segura et al. used an age–cohort model to predict mortality from pleural mesothelioma in the Netherlands, estimating that it would peak in 2017 and decrease thereafter [11]. Price and Ware predicted the incidence of MM in the US using an age–cohort model and found that the crude incidence in men would peak between 2000 and 2004 [12]. Pitarque et al. projected MM mortality from 2007 to 2016 in Spain using the APC model and predicted that it would continue to increase until at least 2016 [13]. Mensi et al. projected Italy’s incidence of MM using an age–cohort model and predicted that the peak would occur around 2019 [14].

Most prediction studies for MM have been conducted in developed countries and used an age–cohort or APC model. However, these predictions did not directly address asbestos consumption or exposure. Several ecological studies have shown a linear relationship between asbestos consumption and MM incidence or mortality [15,16,17,18]. Thus, a regression model using asbestos consumption can also be considered. In 2013, Bahk’s study predicted MM mortality from 2015 to 2070 for each continent using a negative binomial regression model based on asbestos consumption and data on MM mortality from 54 countries [19]. In addition, Hodgson et al. predicted MM mortality in the UK by constructing a Poisson regression model based on each age group’s exposure potential [20]. However, no study has compared an APC model to a regression model in terms of their performance in MM prediction. In particular, because asbestos consumption has changed dramatically over a relatively short period in South Korea, it is necessary to estimate the occurrence of MM using a regression model based on asbestos consumption and to compare the results with previous prediction models.

In this study, we aimed to update our previous predictions [4] of MM in South Korea using the APC model. We also made predictions using a Poisson regression model based on asbestos consumption and verified the predictions against actual incidence data.

## 2. Materials and Methods

### 2.1. Data on MM Incidence

We obtained data on the incidence of MM (C45) for the 1994–2018 period from the Korea Central Cancer Registry (KCCR). MM incidence data for 1999–2018 are freely available on the website of the Korean Statistical Information Service (KOSIS) [21]. The incidence data for 1994–1998 were separately requested from the KCCR, and we received the data after obtaining approval. For the APC model, the incidence data for MM were organized according to sex in 5-year periods.

### 2.2. Data on Asbestos Consumption

We used data on the import and export of asbestos from the Statistical Yearbook of Foreign Trade published by the Korea Customs Service (KCS). The KCS Yearbook collected statistics on the import and export of asbestos along with data for other minerals until 1975; hence, asbestos could not be classified separately before then. However, KCS data on asbestos from 1976 were available. We also extracted data on asbestos production from 1976 to 1990 from the Statistical Yearbook provided by the Korea Mining Promotion Corporation. Previous import, export, and production data were extracted from the Mineral Yearbook provided by the US Geological Survey (USGS) [22]. The USGS provides statistics on the import, export, and production of asbestos in each country every 5 or 10 years. For South Korea, it provided statistics on the import, export, and production of asbestos for 1940, 1950, 1960, 1970, and 1975. We extrapolated asbestos imports, exports, and production for 1941–1949, 1951–1959, 1961–1969, and 1971–1974, assuming a linear relationship between periods of 5 or 10 years. We defined asbestos consumption as the amount produced plus the amount imported and minus the amount exported. The annual asbestos import, export, and production statistics are presented in Supplementary Table S1.

### 2.3. Data on Population

As population data, we used the annual resident population data (1994–2018) and projected population data (2019–2038) from KOSIS [23,24]. For the APC model, we organized the population data into 5-year groups. For the Poisson regression model including asbestos consumption, however, we used annual data rather than organizing the data into 5-year groups.

### 2.4. APC Model

We first used Møller’s APC model with power-link function to estimate future incidence rates. Møller’s APC model is widely used for predicting cancer incidence. The International Agency for Research on Cancer used this model to predict the future global cancer burden based on historical national incidence data [25]. It has also been used for our previous predictions. The model is written as follows [26]:

*R_ap_* = (*A_a_* + *D*·*p* + *P_p_* + *C_c_*)^5^

where:

*R_ap_*: Incidence rate for age group *a* in calendar period *p*;
*A_a_*: Age component for age group *a*;
*D*: Common drift parameter which summarizes the linear component of trend;
*P_p_*: Non-linear period component of period *p*;
*C_c_*: Non-linear cohort component in cohort *c*.

We performed analyses using the NORDPRED R package [27] based on the APC model.

### 2.5. Poisson Regression Model Based on Asbestos Consumption

We constructed a MM prediction model based on cumulative asbestos consumption and used a Poisson regression model to predict the incidence of MM due to asbestos exposure. The latent period for MM was set at 40 years, and cumulative asbestos exposure was defined as the amount of lifetime asbestos exposure before the latent period. Cumulative asbestos exposure was calculated as:

$${\left[\left(\overset{Y-Lt}{\underset{Y-a}{\sum }}C\right)\cdot e/d\right]}_{a,\;Y}$$

The prediction model is written as follows:

$$\log\left(M_{a,Y}\right)=\beta_{0}+\beta_{1}{\left[\left(\overset{Y-Lt}{\underset{Y-a}{\sum }}C\right)\cdot e/d\right]}_{a,Y}+\beta_{2}Sex+\beta_{i1}\sum {Age}_{i}+\beta_{i2}\sum Sex\cdot Age_{i}+\varepsilon_{a,Y}$$

where:

*a*: Age group (0–39, 40–49, 50–59, 60–69, 70–79, 80+);
*Y*: Year of occurrence;
*M_a,Y_*: Age-specific incident cases of MM for each year;
*C*: Asbestos consumption per capita (kg) for each year;
*d*: Data available years for each age group;
*e*: Exposed year (from birth to occurrence, with latency);
*Sex*: Dummy variable of sex (0: women, 1: men);
*Age_i_*: Dummy variables of age group for each age group (baseline: 0–39 years of age);
*Sex*·*Age_i_*: Interaction variables for sex and age;
*L_t_*: Latent period, 40 years;
*β*_0_: Intercept;
*β*_1_*,β*_2_*, β_i_*_1_*, β_i_*_2_: Regression coefficients;
ε*_a,Y_*: Errors.

We used SAS version 9.4 (SAS Institute Inc., Cary, NC, USA) to run the Poisson regression model.

### 2.6. Statistical Analysis

We calculated the predicted incidence of MM for 2014–2018 using Møller’s APC model, and a Poisson regression model based on MM incidence data for 1994–2013. We compared the expected number (E) of MM cases predicted by each model and the actual observed numbers (O) of MM incidence data for 2014–2018, and calculated the E/O ratio and corresponding 95% confidence intervals (CIs) for empirical validation. The 95% CIs of the E/O ratio were calculated using the Poisson method. This method assumes that the observed number of cases follows a Poisson distribution, and calculates the 95% CIs of the E/O ratio using a normal approximation to the Poisson distribution [28,29]. The formula for calculating the 95% CIs of the E/O ratio is as follows:

$$\frac{E}{O}\cdot \exp\left(\pm 1.96\sqrt{\frac{1}{O}}\right)$$

We also predicted the incidence of MM for 2014–2018 according to age group, and calculated the E/O ratio and corresponding 95% CIs by comparison with the observed incidence. We selected the model for which the E/O ratio was closer to 1 as a final prediction model, as it was considered more reliable. Then, we calculated the predicted crude incidence of MM from 2019 to 2038 using the final prediction model.

## 3. Results

### 3.1. Prediction for 2014–2018 and Empirical Validation

Based on the incidence of MM for 1994–2013, Møller’s APC model estimated that 461 and 254 cases of MM would occur over the next 5 years (2014–2018) in men and women, respectively. The Poisson regression model based on asbestos consumption estimated that 634 new cases of MM would occur in men over the next 5 years (2014–2018), and 351 would occur in women. The actual incidence of MM in men and women from 2014 to 2018 was 501 and 233 cases, respectively (Table 1). The number of incident cases in men was greater than the number predicted by the APC model and lower than that predicted by the Poisson regression model. In women, the actual number of incident cases was lower than the numbers predicted by both models. The E/O ratios of the APC model for both men and women were closer to 1. Moreover, the corresponding 95% CIs of the E/O ratios included 1, indicating that the number predicted by the APC model was not significantly different from the actual number of MM incident cases. In addition, when comparing the predicted numbers for each age group, the Poisson regression model overestimated the incidence of MM in the 40–59-year age group relative to the actual occurrence (Table 2). Thus, the accuracy of the APC model can be considered better and its predictions more reliable. Therefore, we selected the APC model as our final prediction model.

### 3.2. Predictions for 2019–2038 Based on the APC Model

Based on the last 20 years (1999–2018), we predicted the incidence of MM over the next 20 years (2019–2038) using Møller’s APC model, which was selected as the final prediction model. This model predicted that 3610 and 1445 new cases of MM in men and women, respectively, will occur in the next 20 years (2019–2038) (Table 3).

**Figure 1:**
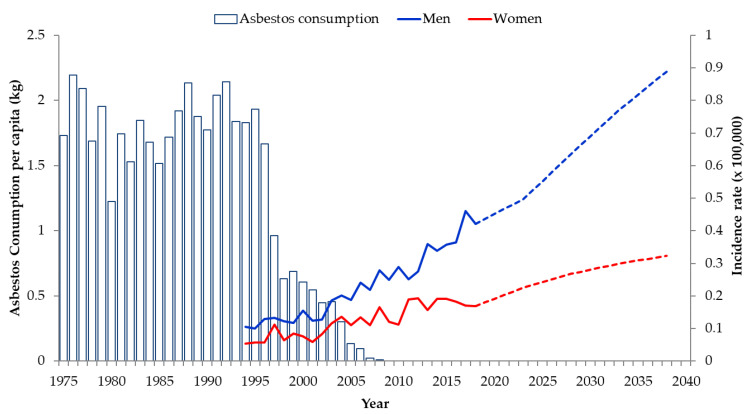
Asbestos consumption during the period 1975–2008 and observed (1994–2018) and predicted (2019–2038) crude incidence rates of malignant mesothelioma.

In both men and women, the number of MM cases was predicted to increase until 2034–2038. However, the increasing trend was slightly different for men and women. In men, the incidence rate was expected to increase more than twice as fast until 2034–2038, whereas it was predicted to increase more slowly in women (Figure 1).

## 4. Discussion

In this study, the incidence of MM in South Korea was predicted to continuously increase until 2038. We first predicted the incidence of MM using an APC model and Poisson regression model, and then compared the predicted values with actual incidence data. The Poisson regression model predicted much higher numbers of MM cases than the APC model. We judged that the values predicted by the Poisson regression model were overestimates, concluded that the APC model was more reliable, and thus selected the APC model as the final model to predict the incidence of MM over the next 20 years. According to the APC model, the crude incidence rate was predicted to increase continuously and sharply in men. In women, the crude incidence rate was also predicted to increase continuously, but the rate of increase was slower compared to that for men, and the predicted increase in the number of cases was smaller.

This was a follow-up of our previous study [4], which predicted the incidence of MM until 2033 in Korea for the first time using the APC model. Our previous analysis had some limitations. The available data on MM incidence were from 1994, but the early incidence data on KCCR had problems with reliability. Population-based regional cancer registry programs, which comprise the current cancer registration system, started in 1991 in Seoul and then expanded to other regions in 1999 [30]. For regions outside Seoul, data before 1999 came from hospital-based registries. Because the hospital-based registries were passive registration systems, the number of incident cases might have been underreported, influencing the predictions. In addition, the range of available data was limited, so the model predictions could not be compared with actual incidence data. Furthermore, the amount of asbestos consumption was not considered in the previous prediction model. In the present study, we addressed these limitations of the 2017 analysis and updated the scope of future predictions. We added incidence data for the past 5 years, which were released since the previous study was conducted, and updated our predictions. By adding the latest data, we could exclude the initial cancer incidence data that reduced the reliability of the model. We also validated the model by comparing the predicted incidence with the actual incidence data even though the validation period was short and limited. In addition, we constructed a Poisson regression model that included asbestos consumption for additional prediction and compared the results of the two models with actual incidence data to achieve a more accurate prediction model.

The previous analysis in 2017 using the APC model predicted that 2380 and 1199 new cases of MM would occur in men and women, respectively, between 2014 and 2033. In the present study, 3610 and 1445 new cases were predicted to occur in men and women, respectively, over the next 20 years (2019–2038); this is 1.5 times more than the number derived from the previous analysis in men and slightly more than the number estimated for women. In addition, in the previous analysis, the incidence of MM was predicted to increase continuously until 2033 in men, but in women, the incidence was expected to peak in 2024–2028 and decrease thereafter. The present analysis, however, predicted that the incidence of MM would continue to increase until 2038 in both men and women. In addition, in the previous analysis, the crude incidence rate and age-standardized incidence rate were presented together, whereas only the crude incidence rate was employed in the present study. In the previous study, the patterns of crude incidence and age-standardized incidence differed greatly. This was due to the high risk of age-specific incidence in older age groups because of the long latent period and cumulative exposure to asbestos. More people exposed to asbestos appear to develop MM as they age and pass through the latent period, so it is not meaningful to present the age-adjusted incidence, and the incidence risk can be underestimated. Thus, in this study, we analyzed and presented only the number of cases and the crude incidence rate.

In the present analysis, it seems that the predictions for men and women differed mainly due to differences in the pathway of asbestos exposure. MM in men is largely caused by occupational exposure, with few cases likely caused by environmental exposure. By contrast, in women, MM is thought to be mainly caused by environmental exposure. Environmental asbestos exposure varies by period but is persistent, so it has a periodic effect, and occupational exposure exhibits a cohort effect because it occurs mainly in the age group exposed only at a specific time when asbestos was used. Thus, MM in men is considered a result of a combination of the cohort effect and period effect, whereas MM in women is considered a result of the period effect. In other words, due to a cumulative period effect, MM will likely continue to be caused by environmental exposure to asbestos in both men and women. In the male cohort who experienced occupational exposure from the 1980s to the mid-1990s, when asbestos was commonly used, MM would develop in earnest after the latent period. Thus, it was predicted that many more cases of MM would occur in men. We also estimated the incidence of MM by creating a Poisson regression model that included age-specific cumulative asbestos exposure potential. A comparison of the numbers of MM incident cases predicted by the APC and Poisson regression models with the actual number of incident cases from 2014 to 2018 revealed that the figure predicted by the APC model was closer to the actual number of incident cases. We considered the Poisson regression model as a model intended to compensate for the shortcomings of the previous prediction model, which did not use information on asbestos consumption, but the actual predictions did not more closely reflect reality.

The low predictive accuracy of the Poisson regression model can be attributed to many assumptions related to asbestos exposure. Asbestos consumption is an important factor in predictions using the Poisson regression model, and the assumptions that determine cumulative exposure, such as the lag period, asbestos exposure potential, and asbestos clearance, have a large influence. Our Poisson regression model assumed a latent period of 40 years, calculated asbestos exposure potential by dividing age groups into 10-year units, and did not consider lung clearance. Because the age-specific asbestos potential, latent period, and asbestos clearance values were arbitrary, the prediction results will not be robust if the assumptions change. Moreover, we assumed that all asbestos consumption reflected the exposure level, so more exposure might have occurred. In particular, it is more likely that men were exposed to asbestos than women, but we assumed that individuals encountered the same degree of asbestos exposure regardless of sex. As a result, the female MM incidence predicted by the Poisson regression model differed from that predicted by the APC model.

In addition, the incidence in the 40–59-year age group predicted by the Poisson regression model was much higher than the actual incidence, whereas the predicted incidence at 80 years of age or older was lower. Because the latent period was assumed to be 40 years, cumulative exposure from 1974 to 1978 was predicted to be associated with the occurrence of mesothelioma from 2014 to 2018. In the case of the 40–59-year age group, exposure at a young age was thought to cause MM, but in fact, the people who had been exposed to asbestos in the 1970s were in their 20s to 50s, the economically active population at that time. The Poisson regression model did not sufficiently reflect this cohort effect, and the accuracy of the model was degraded due to overestimation in the middle-aged group.

However, even if the predictive power of the Poisson regression model is low at the present time, its future predictive capability may differ depending on the model structure and the prediction period. Therefore, it is necessary to pay attention to these aspects while updating the model in the future.

Among the studies in other countries using the APC model or the age–cohort model mentioned in the Introduction, the time points with the predicted peak values are now in the past when viewed in the present time. We can thus validate the previous predictions in some countries by comparing them with actual MM incidence or mortality data. In the UK, the number of MM deaths was predicted to continue to increase in men until 2020, but it actually peaked in 2014–2016 and then decreased [31]. In Denmark, the incidence of MM was expected to peak in 2015, but the actual incidence has continued to increase to date [32]. In the Netherlands, the incidence was expected to peak in 2017 and decrease thereafter, and the actual incidence peaked in 2016, similar to the forecast [33]. In the US, the number of cases in men was expected to peak in 2000–2004, whereas the actual crude incidence peaked in 2005 [34]. Although there were some differences, the peak time points of MM incidence or mortality in other countries predicted by the APC or age–cohort model were somewhat similar to the actual MM statistics.

Although our APC model did not directly consider the amount of asbestos used, we represented the temporal relationship between changes in asbestos exposure and the predicted incidence of MM graphically (Figure 1). Although asbestos use in South Korea peaked in the early 1990s, our prediction showed that the incidence of MM will continue to increase for 45 years after the peak use. This is also likely to be the case in other countries. Asbestos use peaked in the US in around 1950, and in the UK around 1960 [35], but the incidence of MM only started to decrease after 55 years in both countries. Denmark banned the use of asbestos in 1986 [36], but MM continues to occur. The reason why the interval between peak asbestos use and peak MM incidence exceeds the latent period of MM is thought to be due to continuous exposure from buildings or facilities built using asbestos, and to environmental exposure to naturally occurring asbestos after the peak or ban of asbestos use.

There are some limitations to this study. First, as with our previous prediction, the APC model selected as the final prediction model estimated the incidence of MM assuming that past trends would be reflected in future trends. Therefore, there may be differences in the actual incidence of MM because unexpected occurrences in the future were not considered. The population structure is an important factor in APC modeling [37], and since our predictions used the projected population structure from KOSIS, they may be affected by changes in various unpredictable demographic factors in the future. Next, early cases of MM have the potential for misdiagnosis. As mentioned earlier when describing the limitations of previous studies, MM incidence records before 1999 were kept in hospital-based registries, so there is a high probability of missing cases. When predicting the occurrence from 2019 to 2038, most of the early incidence data were excluded, in contrast with our 2017 study, but during validation of the predictions, an error resulted in the inclusion of these early incidence data. Finally, as previously mentioned, since the Poisson regression model in the present study is based on multiple assumptions, it is not robust and has limitations in comparing and verifying the prediction models.

Despite these limitations, we here report the only quantitative predictions of MM incidence in South Korea based on updating our previous model. In addition, we constructed a Poisson regression model based on asbestos consumption as well as an APC model and then compared the values predicted by the two models. Verification was conducted through comparison with actual incidence data, although data were limited.

## 5. Conclusions

This study predicts that the incidence of MM will increase continuously in both men and women over the next 20 years. Compared to our previous predictions, the current study predicted a greater number of MM cases overall and a different incidence trend in women. Because the prediction model is based on several assumptions, it is inevitably accompanied by uncertainty. Therefore, it is necessary to update the prediction of MM occurrence at regular intervals to reduce the uncertainties associated with the current predictions.

## Figures and Tables

**Table 1:** Observed number of malignant mesothelioma cases during the 1994–2018 period and comparison with the number of cases predicted using the age–period–cohort (APC) model and Poisson regression model for 2014–2018.

Sex	Observed Cases					Predicted Cases for 2014–2018		E/O Ratio (95% CI) *	
	1994–1998	1999–2003	2004–2008	2009–2013	2014–2018	APC Model	Poisson Model	APC Model	Poisson Model
Men	137	171	277	361	501	461	634	0.920 (0.843–1.004)	1.265 (1.159–1.381)
Women	79	100	161	195	233	254	351	1.090 (0.999–1.119)	1.506 (1.380–1.644)

* E: expected number of cases, O: observed number of cases, CI: confidence interval.

**Table 2:** Comparison by age group (years) of malignant mesothelioma cases predicted using the APC model and Poisson regression model with observed cases during the 2014–2018 period.

Age Group	Men					Women				
	Observed Cases	Predicted Cases		E/O Ratio (95% CI) *		Observed Cases	Predicted Cases		E/O Ratio (95% CI) *	
		APC Model	Poisson Model	APC Model	Poisson Model		APC Model	Poisson Model	APC Model	Poisson Model
0–39	11	11.6	17.8	1.055 (0.966–1.151)	1.618 (1.483–1.766)	15	19.7	13.5	1.313 (1.203–1.434)	0.9 (0.825–0.982)
40–49	25	38.8	95.4	1.552 (1.422–1.694)	3.816 (3.496–4.165)	20	13.8	45.9	0.69 (0.632–0.753)	2.295 (2.103–2.505)
50–59	95	95	275.1	1 (0.916–1.092)	2.896 (2.653–3.161)	50	61.6	132.1	1.232 (1.129–1.345)	2.642 (2.420–2.884)
60–69	165	121	141.0	0.733 (0.672–0.800)	0.855 (0.783–0.933)	60	76.8	87.4	1.28 (1.173–1.397)	1.457 (1.335–1.590)
70–79	157	163.2	101.5	1.039 (0.952–1.135)	0.646 (0.592–0.706)	61	50.4	56.3	0.826 (0.757–0.902)	0.923 (0.846–1.007)
80+	48	31.3	12.0	0.652 (0.597–0.712)	0.25 (0.229–0.273)	27	31.2	16.0	1.156 (1.059–1.261)	0.593 (0.543–0.647)
	501	460.9	642.7	0.920 (0.843–1.004)	1.265 (1.159–1.381)	233	253.6	351.3	1.090 (0.999–1.119)	1.506 (1.380–1.644)

* E: expected number of cases, O: observed number of cases, CI: confidence interval.

**Table 3:** Observed (1999–2018) and predicted cases (2019–2038) of malignant mesothelioma.

Sex	Observed				Predicted			
	1999–2003	2004–2008	2009–2013	2014–2018	2019–2023	2024–2028	2029–2033	2034–2038
Men	171	277	361	501	645	825	999	1141
Women	100	161	195	233	291	348	389	417

## Data Availability

The numbers of incident cases for malignant mesothelioma by sex and age group from 1999 to 2018 in South Korea were obtained from the Korean Statistical Information Service (KOSIS) at https://kosis.kr (accessed on 17 February 2021). The numbers of incident cases before 1999 can be used after deliberation and approval by the Korea Central Cancer Registry (KCCR). The population data of South Korea are also available at https://kosis.kr (accessed on 19 February 2021). Information on the amount of asbestos consumption, which is utilized in this study, is presented in Supplementary Materials.

## Supplementary Materials

The following are available online at https://www.mdpi.com/article/10.3390/ijerph18126614/s1, Supplementary Table S1: Amounts of asbestos imported, exported, and produced in South Korea by year.
