# Supplementary material for: Future Incidence of Malignant Mesothelioma in South Korea: Updated Projection to 2038
Source: Int J Environ Res Public Health. 2021 Jun 19;18(12):6614. doi: 10.3390/ijerph18126614 (PMC8296497; doi:10.3390/ijerph18126614)
Supplement: Supplementary file 1 [file ijerph-18-06614-s001.zip › ijerph-1226680-supplementary.pdf]

Supplementary Table S1. Amounts of asbestos imported, exported, and produced in South Korea by year (unit: metric ton)

Year	Import	Export	Production	Year	Import	Export	Production	Year	Import	Export	Production
1940	5,081	–	508	1963	–	–	2,037	1986	68,017	0	2,983
1941	–	–	–	1964	–	–	1,402	1987	77,598	41	2,518
1942	–	–	–	1965	–	–	1,710	1988	87,470	0	2,428
1943	–	–	–	1966	–	–	687	1989	77,475	0	2,351
1944	–	–	4,815	1967	–	–	2,388	1990	74,549	5	1,534
1945	–	–	1,303	1968	–	–	–	1991	88,753	157	0
1946	–	–	–	1969	–	–	6,515	1992	95,476	23	0
1947	–	–	–	1970	35,292	–	1,513	1993	82,854	18	0
1948	–	–	–	1971	–	–	–	1994	83,276	86	0
1949	–	–	–	1972	–	–	–	1995	88,722	16	0
1950	–	–	46	1973	–	–	–	1996	77,145	15	0
1951	–	–	46	1974	–	–	–	1997	44,985	0	0
1952	–	–	46	1975	56,960	–	4,345	1998	29,619	1	0
1953	–	–	46	1976	74,206	16	4,762	1999	32,519	0	0
1954	–	–	46	1977	70,255	75	6,180	2000	28,972	11	0
1955	–	–	66	1978	48,898	10	13,616	2001	26,307	5	0
1956	–	–	54	1979	58,610	8	14,804	2002	21,503	9	0
1957	–	–	96	1980	36,787	30	9,854	2003	22,032	0	0
1958	–	–	22	1981	53,787	40	13,614	2004	14,580	0	0
1959	–	–	88	1982	44,038	12	15,933	2005	6,476	0	0
1960	–	–	740	1983	60,896	0	12,506	2006	4,701	0	0
1961	–	–	341	1984	59,693	0	8,062	2007	1,094	0	0
1962	–	–	1,333	1985	57,143	17	4,703	2008	383	0	0
Total									1,796,152	595	146,061
